# Contagious bovine pleuropneumonia: Seroprevalence and risk factors in Western Oromia, Ethiopia

**DOI:** 10.4102/ojvr.v83i1.958

**Published:** 2016-05-12

**Authors:** Garuma Daniel, Mukarim Abdurahaman, Getachew Tuli, Benti Deresa

**Affiliations:** 1 School of Veterinary Medicine, Jimma University, Ethiopia; 2 National Animal Health Diagnostic and Investigation Center, Sebeta, Ethiopia

## Abstract

Contagious bovine pleuropneumonia (CBPP) is one of the most important threats to cattle health and production in Ethiopia. At the livestock farm of the Bako Agricultural Research Center, an outbreak of respiratory disease of cattle occurred in May 2011, and many animals were affected and died before the disease was diagnosed. Therefore, this study was designed to determine the seroprevalence of CBPP antibodies in selected districts of Western Oromia Region and to assess the potential risk factors for the occurrence of the disease. A cross-sectional study was conducted from November 2013 to March 2014 in three selected districts of Western Oromia Region. A total of 386 sera were examined for the presence of specific antibodies against *Mycoplasma mycoidesmycoides* small colony (MmmSC), using a competitive enzyme-linked immunosorbent assay. The risk factors that were evaluated in this study were geographical location, age, sex, breed and body condition. The overall seroprevalence in this study was 28.5%. The seroprevalence of *Mycoplasma mycoidesmycoides* small colony antibodies at the district level was 40.3%, 19.0% and 5.7% in Gobbu Sayyo, BakoTibbe and Horro districts, respectively. There was a statistically significant variation (*p* < 0.05) in the prevalence of antibodies amongst the districts. However, animal-related risk factors, such as age, sex, breed and body condition, were not significantly associated (*p* > 0.05) with the serological status of the animal. This study showed that the overall prevalence of CBPP in Western Oromia Zones was high. This warrants the implementation of appropriate preventive and control measures to minimise the economic losses associated with the disease.

## Introduction

Contagious bovine pleuropneumonia (CBPP) is an acute, subacute or chronic respiratory disease of cattle caused by *Mycoplasma mycoidesmycoides* small colony (MmmSC) (OIE 2008). It is now one of the most important transboundary diseases, along with foot and mouth disease (FMD), although its clinical effects on animals are far more severe than foot and mouth disease (Nicholas, Ayling & McAuliffe 2008). The disease is endemic to parts of Africa, having been eradicated elsewhere. In almost all African countries, it is a notifiable disease and there are official controls on the import of cattle. However, in many countries there are nomadic people who move from country to country, for example the Fulani in West Africa and the Maasai in East Africa. Wars, famine as well as inadequate financing of veterinary services have resulted in CBPP spreading widely in East and Central Africa (Radiostits *et al*. 2006). After rinderpest was eradicated, CBPP has become the most important cattle disease that hinders livestock development in Ethiopia.

According to the OIE (2008), the highest numbers of cases or outbreaks reported in 20 countries were recorded in Ethiopia. Although there has been no comprehensive epidemiological investigation into the distribution and impact of CBPP in the country, it is considered the most important cattle disease in especially pastoral and agro-pastoral areas.

In Western Oromia, where mixed farming is the mainstay of the communities, cases of animal diseases of unknown aetiology are often reported. These diseases directly affect livestock production and productivity, consequently threatening the livelihood of small-scale farmers in the area. At the livestock farm of the Bako Agricultural Research Center, an outbreak of respiratory disease of cattle occurred in May 2011, and many animals fell sick and died before the disease was diagnosed. According to the outbreak investigation carried out by the National Animal Health Diagnostic Investigation Centre, based on serology, bacterial culture and postmortem examination the cause of the outbreak was confirmed to be MmmSC, the aetiological agent of CBPP. For further verification, serum samples were taken from all cattle older than 6 months of age twice at 2-month intervals. The results revealed that almost all animals in the Center were seropositive.

No previous investigation has been carried out to determine the prevalence of CBPP in this zone. Subsequently, the Center was advised not to allow movement of any cattle from the Center until the problem was addressed and further CBPP surveillance was carried out to determine the presence and distribution of the disease in the area. The present study was conceived against this background to determine seroprevalence and identify risk factors for the occurrence of CBPP in the study areas.

## Materials and methods

### Description of the study area

**FIGURE 1:**
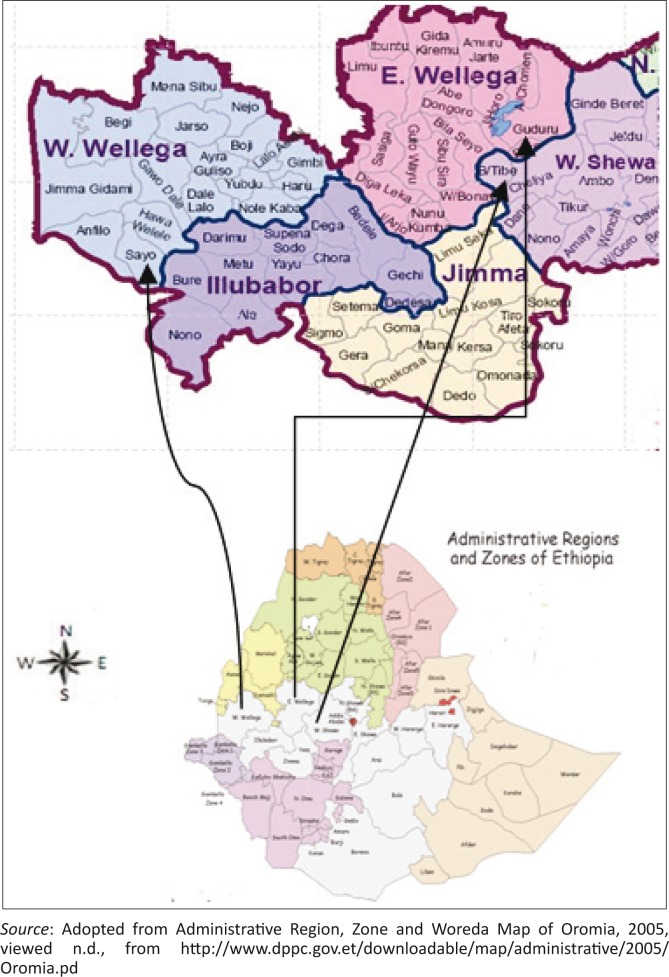
Map showing the location of the study area.

The study was carried out in selected districts of three Western Oromia Zones (Western Shoa, HorroGuduruWollegga and Eastern Wollegga) (Figure 1). The BakoTibbe district of Western Shoa is located in the western part of Ethiopia, 238 km from Addis Ababa, the capital of Ethiopia. The district has an altitude ranging from 1300 m a.s.l. to 2998 m a.s.l and the average annual temperature ranges from 13.7 °C to 27.8 °C. The livestock population of the area includes 137 343 cattle (BTWOARD 2012). The Gobbu Sayyo district of Eastern Wollegga is located 281 km west of Addis Ababa at an altitude of 1650 m a.s.l. The temperature of the area ranges from 13.6 °C to 28.8 °C. This district has 226 791 cattle (GSWOARD 2011). Horro district is located in the HorroGuduruWollegga Zone, 314 km west of Addis Ababa at an altitude of 2430 m a.s.l. The temperature of the area ranges from 10.8 °C to 22.3 °C. The district has 362 507 cattle (HWRDO 2009).

### Study population

The study population comprised cattle of both local (zebu) and cross-breeds in the three zones.

### Study design

A cross-sectional study was carried out in the three above-mentioned zones of the Oromia Regional State. Three districts (BakoTibbe, GobbuSayyo and Horro) were purposively selected based on their linkage with the Bako Agricultural Research Center.

### Sampling frame and sample size determination

The sampling frame consisted of a list of districts and associated cattle populations in the selected areas. The human population in each village could not be ascertained. The sampling methods were based on CBPP status, namely, outbreak area, suspected area and free area. Because the previous prevalence of the disease in the region was not known, 50% expected prevalence and a 5% absolute level of precision was considered to calculate the number of animals to be sampled (Thrusfield 1995) as follows:

$$N=\frac{1.96^{2}\times P_{\exp}(1-P_{\exp})\times 100}{d^{2}}$$ [Eqn 1]

Where *N* = required sample size; *P*_exp_ = expected prevalence; *d* = desired absolute precision.

$$N=\frac{1.96^{2}\times 0.5(1-0.5)\times 100}{0.05^{2}}=384$$ [Eqn 2]

### Sample collection

Animals were restrained by owners and 10 mL of blood was collected from the jugular vein using vacutainer tubes. The samples were kept in the shade in a slanted position for 24 h, after which the sera were transferred to serum tubes and kept at -20 °C until they were tested. Corresponding to each sample, the sample code, age, breed, body condition, site and sex of every animal were recorded.

### Serological testing

Competitive enzyme-linked immunosorbent assay (ELISA) was conducted as recommended by CIRAD-UMR15 (France) and is based on a monoclonal anti-MmmSC antibody named Mab 177/5 as previously described (LeGoff & Thiaucourt 1998). The sensitivity and specificity of the diagnostic test used were, respectively, 98% and 97%. Serum samples were mixed with specific monoclonal antibody (Mab 117/5) in a dilution plate and incubated with gentle agitation for 37 °C for 1 h, then transferred into the MmmSC-coated microplate. After washing, anti-mouse IgG serum–conjugated horse radish peroxidase was added. After a series of washings, the horse radish peroxidase substrate (TMB) was added forming a blue compound that turned yellow when the reaction was stopped. The optical density was read in an ELISA reader at 450 nM and the cut-off point was calculated to validate the results. All sera with percentage inhibition (PI) > 50% were considered positive. Sera with PI between 40% and 50% were considered doubtful and those sera with PI less than 40% were negative.

### Data analysis

The collected data were recorded in a Microsoft Office Excel 2007 spreadsheet. Statistical analyses were performed using SPSS version 20 software. The overall seroprevalence of CBPP was determined using descriptive statistics. Seroprevalence was calculated by dividing the number of positive test results by the total number of animals tested. The chi-square test was used to determine the association between explanatory variables (risk factors) and the dependent variable (serological status of the animals). In all analyses, a confidence level of 95% and *p*-value of 0.05 were applied to determine statistical significance.

## Results

### Prevalence of antibodies against MmmSC using competitive ELISA

The overall seroprevalence of CBPP in the study area was 28.5%. The highest seroprevalence (40.3%) was observed in GobbuSayyo district of Eastern Wollegga Zone, whilst the lowest seroprevalence (5.7%) was recorded in Horro district of HorroGuduruWollegga Zone. There was a statistically significant variation (*p* < 0.05, χ^2^ = 64.13) in seroprevalence among the three districts (Table 1).

**TABLE 1:** Seroprevalence of contagious bovine pleuropneumonia in individual animals in the western part of Oromia, Ethiopia.

District	Number of cattle tested	Number of cattle positive	% positive samples	95% Confidence interval	χ^2^ (*p*-value)
BakoTibbe	100	19	19	11.8–28.06	−64.13 (0.001)
Horro	70	4	5.7	1.5–14.00	
GobbuSayyo	216	87	40.3	33.6–47.1	

**Total**	**386**	**110**	**28.5**	**24.04–33.2**	**-**

Among the seven villages sampled, the prevalence was highest (58.8%) in Kejo in the GobbuSayyu district. The lowest prevalence (4.8%) was recorded in Lakkuin in the Horro district. There was statistically significant variation (*p* < 0.05, χ^2^ = 73.73) in seroprevalence among the seven villages. The prevalence figures of the seven villages are shown in Table 2.

**TABLE 2:** Seroprevalence of contagious bovine pleuropneumonia in individual animals in the sampled villages.

Site (villages)	Number of animals tested	Number testing positive	% positive	95% Confidence interval	χ^2^ (*p*-value)
Ongobbo	67	27	40.2	28.4–52.99	73.73 (0.001)
Kejo	51	30	58.8	44.1–72.4	
Abba Korra	98	30	30.6	21.6–40.7	
Gitilo	29	2	6.8	0.84–22.7	
Lakku	41	2	4.8	0.59–16.5	
SadanQitte	52	5	9.6	3.1–21.02	
DembiDima	48	14	29.01	16.9–44.06	

**Total**	**386**	**110**	**28.4**	**24.04–33.2**	-

No statistically significant associations were found between the host demographics (breed, age, sex and body condition) and the serological status of the animals (*p* > 0.05) (Table 3).

**TABLE 3:** Seroprevalence of contagious bovine pleuropneumonia measured against different host-related risk factors.

Variables	Number tested	Number positive	% positive	95% Confidence interval	χ^2^ (*p*-value)
**Sex**					0.683 (0.407)
Male animals	142	44	30.9	23.5–39.2	
Female animals	244	66	27.04	21.5–33.08	
**Age**					0.857 (0.409)
Young	133	34	25.5	18.3–33.8	
Adult	253	76	30.3	24.4–36.0	
**Breed**					1.802 (0.209)
Local breed	343	94	27.4	22.7–32.4	
Cross-bred animals	43	16	37.2	22.9–53.2	
**Body condition**					4.384 (0.114)
Poor	113	26	23	15.6–31.8	
Good	91	33	36.2	26.4–47.00	
Medium	182	51	28.02	21.6–35.1	

**Total**	**386**	**110**	**28.4**	**24.04–33.28**	-

## Discussion

Based on the serological results of this study, CBPP was a major cattle health problem in the western zones of the Oromia Region. In this study, a total of 386 serum samples were tested from the three zones and the overall seroprevalence of CBPP was 28.5% (confidence interval = 24.04–33.2). This result is similar to those of Regassa (2001) and Desta (1998), who reported seroprevalences of 28% in the Bodji district of Western Wollegga and 32.5% in western Ethiopia, respectively. However, the overall seroprevalence was lower than 39% reported by Gedlu (2004) in Somali Regional State and 56% reported by Dejene (1996) in North Omo, western Ethiopia, but higher than many others previously reported: 9.4% in Borena (Ahmed 2004), 9.7% in south-western Kenya (Schnier *et al*. 2006), 9.1% in northwest Ethiopia (Gashaw 1998), 16% in Kajiado district, Kenya (Matua-Alumira *et al*. 2006), and 4% in and around Adama, central Ethiopia (Kassaye & Molla 2013). The variation in prevalence reported from different parts of Ethiopia and other countries may be because of differences in agro-ecological systems, cattle management and production systems, population density and the types of tests used to determine the seroprevalence.

The highest prevalence, reported in Gobbu Sayyo, could be related to the presence of larger herds and communal grazing areas, making contact with infected animals more likely. Similar explanations were also given in a study in the Somali Region (Gedlu 2004). The highest herd seroprevalence was observed in Mieso district (100%), followed by Qabribeyah (75%) and Afdem (71.4%). In Western Gojam and Awi Zone (Gashaw 1998), the highest seroprevalence was observed in Banja district (66.3%), followed by Dangila (41.7%) and Denbecha (33.3%).

The prevalence of infection was 30.9% in male and 27.04% in female animals; there was no statistically significant difference (*p* > 0.05) in the occurrence of the disease based on sex. This finding does not concur with that of Schnier *et al*. (2006), who reported a significantly higher prevalence in female animals.

There was no statistically significant variation (*p* > 0.05) in seroprevalence between the local breed (27.4%) and cross-breed (37.2%) cattle or young (25.5%) and adult (30.3%) animals. This result is in close agreement with previous reports by Swai *et al*. (2013) and Matua-Alumira *et al*. (2006) that seropositivity in adults was a bit higher than that in young animals. The small difference in seroprevalence between the two age categories can be attributed to the fact that young animals do not move far away from houses; therefore, there is less chance to come into contact with infected animals. In addition, Masiga, Domenech and Windsor (1995) reported that young animals are more susceptible to acute forms of CBPP than adult cattle and thus acutely infected young animals may die of CBPP and not be available for testing.

The serological test used in this study, competitive ELISA, is more sensitive for detecting chronically infected cattle than other tests. Thus, it is likely that individual animals at the early stage of infection can be missed by the test (Muuka *et al*. 2011; Schubert *et al*. 2011).

## Conclusion

This study documented a high seroprevalence of CBPP in cattle in the western part of Ethiopia, suggesting the disease could cause considerable economic losses through morbidity and mortality. The presence of statistically significant differences in the prevalence of CBPP among the study villages suggests that variation in management factors favours the occurrence and spread of the disease. Therefore, it is recommended, as a short-term intervention, that vaccination with treatment should be started in CBPP-seropositive areas. In the long term, annual vaccination with cattle movement control should be carried out as well as awareness creation among the farmers about the means of transmission of the disease and its high economic importance.
